# Supplementary material for: P-TRAP: a Panicle Trait Phenotyping tool
Source: BMC Plant Biol. 2013 Aug 29;13:122. doi: 10.1186/1471-2229-13-122 (PMC3848748; doi:10.1186/1471-2229-13-122)
Supplement: Additional file 7 — P-TRAP source code. The Netbeans project that contains the source code of the application. [file 1471-2229-13-122-S7.zip › javadoc.zip!/javadoc/org-bioalg-skeletonprocessor/deprecated-list.html]

Deprecated List (SkeletonProcessor)


JavaScript is disabled on your browser.


- Package
- Class
- Tree
- Deprecated
- Index
- Help

*org.bioalg.skeletonprocessor 1.0*

- Prev
- Next

- Frames
- No Frames

- All Classes

# Deprecated API

## Contents

- Package
- Class
- Tree
- Deprecated
- Index
- Help

*org.bioalg.skeletonprocessor 1.0*

- Prev
- Next

- Frames
- No Frames

- All Classes
